# Infective Endocarditis: A Rare Organism in an Uncommon Setting

**DOI:** 10.1155/2012/307852

**Published:** 2012-12-24

**Authors:** Rajiv Ananthakrishna, Ravindranath K. Shankarappa, Naveena Jagadeesan, Ravi S. Math, Satish Karur, Manjunath C. Nanjappa

**Affiliations:** ^1^Department of Cardiology, Sri Jayadeva Institute of Cardiovascular Sciences & Research, Jaya Nagar 9th Block, BG Road, Bangalore, Karnataka 560069, India; ^2^Department of Microbiology, Sri Jayadeva Institute of Cardiovascular Sciences & Research, Jaya Nagar 9th Block, BG Road, Bangalore, Karnataka 560069, India

## Abstract

*Facklamia hominis* is a rare causative organism of infective endocarditis (IE). Only few cases of infection due to *F. hominis* have been reported in the literature. We describe a case of IE due to Gram-positive, alpha-haemolytic, catalase-negative coccus *F. hominis* in an adult patient with rheumatic mitral stenosis. Isolated mitral stenosis is an uncommon valve lesion predisposing to IE. The following paper is being presented to emphasize the possibility of IE due to *F. hominis*, and laboratories need to be alert of the potential significance in appropriate clinical setting.

## 1. Introduction

Infective endocarditis (IE) is one of the major complications in individuals with structural heart disease. The majority of cases of IE are caused by *Streptococci, Staphylococci, Enterococci,* and fastidious Gram-negative *coccobacilli*. In their absence, unusual organisms should be considered. *Facklamia hominis* is one such rare organism [1]. *Facklamia* species are Gram-positive, alpha-haemolytic, catalase negative cocci, which resemble *Streptococcus viridians* on 5% sheep blood agar. Only few cases of infection due to *F. hominis* have been reported in the literature. We present a case of IE due *F. hominis* in an adult patient with rheumatic mitral stenosis. Isolated mitral stenosis is an uncommon valve lesion predisposing to IE. The following paper is being presented to emphasize the possibility of IE due to *F. hominis,* and laboratories need to be alert of the potential significance in appropriate clinical setting.

## 2. Case Report

**Figure 1:**
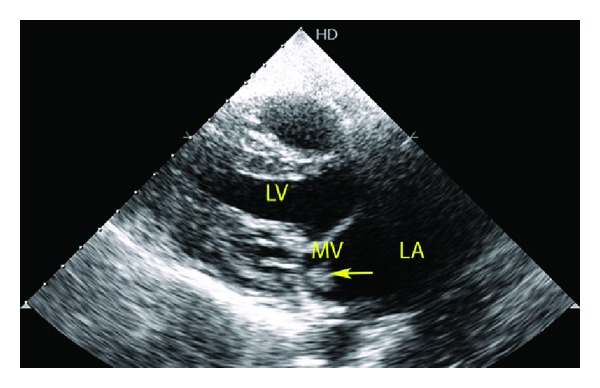
Two-dimensional transthoracic echocardiography in parasternal long axis view illustrating the vegetation (arrow head). In addition, significant submitral pathology can be appreciated. (LA: left atrium, MV: mitral valve, LV: left ventricle).

**Figure 2:**
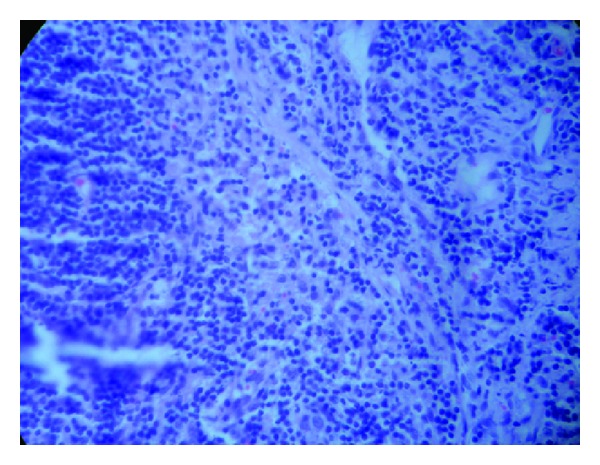
Gram stain from blood culture bottle shows gram-positive cocci in chains.

A 35-year-old male with rheumatic heart disease was admitted to our hospital for evaluation of fever of 3-weeks duration. He had been empirically treated for enteric fever by a primary care physician. The patient had received oral ciprofloxacin for 1 week prior to admission. He was on regular benzathine penicillin prophylaxis and had undergone balloon mitral valvotomy 15 years earlier. The temperature at presentation was 101°F. The patient was hemodynamically stable. Cardiovascular system examination revealed a middiastolic murmur at the apex. The rest of the systemic examination was unremarkable. Three separate sets of blood cultures were obtained for possible IE. Blood cultures were performed using BacT/ALERT^R^ FA bottles (Biomerieux). These bottles were incubated in the Bact/ALERT microbial detection system. Pending culture reports, empirical intravenous treatment with crystalline penicillin (24 million units/24 hr IV, every 4 hr in six equally divided doses) and gentamicin (1 mg/kg IV every 8 hr) was initiated. Results routine blood tests were: haemoglobin 12.2 g/dL, white cell count 16.2 × 10^9^/L (neutrophils: 81%), platelets 226 × 10^9^/L, and erythrocyte sedimentation rate (ESR) 102 mm/1 h. Renal parameters, liver function tests, blood sugars, urine routine, and chest X-ray were normal. Serology for HIV was negative. ECG showed a normal sinus rhythm. Two-dimensional transthoracic echocardiography revealed mobile vegetation attached to the anterior mitral leaflet on the left atrial side (Figure 1). Additional findings include a noncalcified, pliable mitral valve with valve orifice of 1.5 cm^2^, severe submitral fusion and valve gradient of 17/8 mm Hg. There was no associated mitral regurgitation. All the three blood cultures yielded growth within 24 hrs of incubation (Figure 2). The isolate was identified as *F. hominis* by Vitek^R^ 2 system using gram positive identification aids (Reference 21342). The isolate was susceptible to penicillin, amoxicillin, cefotaxime, ceftazidime, ceftriaxone, gentamicin, ciprofloxacin, levofloxain, tetracycline, rifampicin, and vancomycin by the Kirby Bauer method on Muller Hinton Agar with 5% sheep blood, as per Clinical and Laboratory Standards Institute. The patient continued to be febrile ten days after initiation of treatment with crystalline penicillin and gentamicin. There were no complications of IE. Antibiotic treatment was changed from crystalline penicillin to ceftriaxone (2 grams IV, once daily). Intravenous treatment was continued for a period of six weeks (6 weeks of ceftriaxone and 4 weeks of gentamicin). The patient's general condition improved, fever subsided, and ESR reduced to 30 mm/1 h at the end of treatment. Repeat blood culture after completion of antibiotic course yielded no growth.

## 3. Discussion

The microbiology of native valve IE is relatively predictable. *Streptococcus viridans* and *Staphylococcus aureus* are the common causative agents. However, unusual organisms like *F. hominis* cannot be ignored when isolated from multiple blood cultures. IE due to *F. hominis* is a rare entity. Todate, only one case of *F. hominis* endocaritis has been published in the literature [1] and as such, there is a lack of awareness regarding Facklamia species and their clinical relevance. This organism could easily be discarded as contaminant.

*Facklamia* species are gram positive cocci resembling *Streptococcus viridans *on 5% sheep blood agar. Since the first description of the genus *Facklamia* in 1997 [2], several strains of *Facklamia* have been isolated. The species of the genus *Facklamia* described include *F. hominis, F. ignava, F. sourekii, F. languida, and F. tabaciasalis* [3]. With the exception of *F. tabaciasalis*, the other *Facklamia* species have been isolated from human clinical specimens. Clinical source of *Facklamia* species include blood cultures, abscess, bone, cerebrospinal fluid, gall bladder, urine, placenta, and vaginal swab [3–5]. The natural habitat of *Facklamia* species is the female genital tract [3]. Therefore, the majority of clinical isolates are reported from females, and the source of infection and mode of transmission of Facklamia bacteremia in males is uncertain. Further studies on newly diagnosed cases may provide a possible explanation regarding the circumstances predisposing to manifestation of disease in men. Management of systemic infections with *Facklamia* is challenging because of varied susceptibility and resistance patterns. The duration of treatment in IE is unclear. The earlier reported case of *F. hominis* IE had a fatal myocardial infarction 2 days following initiation of intravenous antibiotics [1]. Based on the clinical response, we successfully managed with intravenous ceftriaxone for 6 weeks and gentamicin for 4 weeks.

A positive blood culture is a major criterion for the diagnosis of IE. Hence, early detection of bacteraemia using automated BacT/ALERT^R^ system, as in our report, saves time and points towards diagnosis of IE in suspected individuals. The use of fully automated Vitek^R^ 2 system for accurate identification of organism to species level, compared to traditional biochemical tests, is highlighted. In addition, antibiotic susceptibility is possible, which guides the treating physician for initiation of pertinent antibiotics.

An additional interesting finding in this report is the cardiac lesion predisposing to infective endocarditis. This patient had an isolated rheumatic mitral stenosis, which is an uncommon valve lesion predisposing to IE. The estimated risk of IE in patients with mitral stenosis is 0.17 per 1000 patient years [6]. IE is more likely to occur in mild mitral stenosis with noncalcified pliable leaflets, as documented in our case. Hence, although rare, IE should be considered in the differential diagnosis of prolonged fever in isolated mitral stenosis. The consequence of not considering IE can profoundly influence management and outcome.

To conclude, rare organisms like *F. hominis* should be considered in the microbiological spectrum causing infective endocarditis. Isolates of *Facklamia* species from blood cultures are frequently discarded as contaminants. This paper highlights the potential significance of such isolates in the setting of infective endocarditis.
